# Pre-miR-146a (rs2910164 G>C) Single Nucleotide Polymorphism Is Genetically and Functionally Associated with Leprosy

**DOI:** 10.1371/journal.pntd.0003099

**Published:** 2014-09-04

**Authors:** Paula F. T. Cezar-de-Mello, Thiago G. Toledo-Pinto, Carolinne S. Marques, Lucia E. A. Arnez, Cynthia C. Cardoso, Luana T. A. Guerreiro, Sérgio L. G. Antunes, Márcia M. Jardim, Claudia de J. F. Covas, Ximena Illaramendi, Ida M. Dias-Baptista, Patrícia S. Rosa, Sandra M. B. Durães, Antonio G. Pacheco, Marcelo Ribeiro-Alves, Euzenir N. Sarno, Milton O. Moraes

**Affiliations:** 1 Laboratório de Hanseníase, Instituto Oswaldo Cruz, Fundação Oswaldo Cruz (FIOCRUZ), Rio de Janeiro, Brasil; 2 Instituto Lauro de Souza Lima, Bauru, São Paulo, Brasil; 3 Centro de Ciências Médicas, Universidade Federal Fluminense, Niterói, Rio de Janeiro, Brasil; 4 Programa de Computação Científica, Fundação Oswaldo Cruz (FIOCRUZ), Rio de Janeiro, Brasil; University of Tennessee, United States of America

## Abstract

*Mycobacterium leprae* infects macrophages and Schwann cells inducing a gene expression program to facilitate its replication and progression to disease. MicroRNAs (miRNAs) are key regulators of gene expression and could be involved during the infection. To address the genetic influence of miRNAs in leprosy, we enrolled 1,098 individuals and conducted a case-control analysis in order to study four miRNAs genes containing single nucleotide polymorphism (miRSNP). We tested miRSNP-125a (rs12975333 G>T), miRSNP-223 (rs34952329 *>T), miRSNP-196a-2 (rs11614913 C>T) and miRSNP-146a (rs2910164 G>C). Amongst them, miRSNP-146a was the unique gene associated with risk to leprosy *per se* (*GC* OR = 1.44, p = 0.04; *CC* OR = 2.18, p = 0.0091). We replicated this finding showing that the *C*-allele was over-transmitted (p = 0.003) using a transmission-disequilibrium test. A functional analysis revealed that live *M. leprae* (MOI 100∶1) was able to induce miR-146a expression in THP-1 (p<0.05). Furthermore, pure neural leprosy biopsies expressed augmented levels of that miRNA as compared to biopsy samples from neuropathies not related with leprosy (p = 0.001). Interestingly, carriers of the risk variant (*C*-allele) produce higher levels of mature miR-146a in nerves (p = 0.04). From skin biopsies, although we observed augmented levels of miR-146a, we were not able to correlate it with a particular clinical form or neither host genotype. MiR-146a is known to modulate TNF levels, thus we assessed TNF expression (nerve biopsies) and released by peripheral blood mononuclear cells infected with BCG Moreau. In both cases lower TNF levels correlates with subjects carrying the risk *C*-allele, (p = 0.0453 and p = 0.0352; respectively), which is consistent with an immunomodulatory role of this miRNA in leprosy.

## Author Summary

In spite of the successful drug therapy, leprosy is still affecting people worldwide. It is well known that host genetic background influences leprosy development and that genetic variants have been associated with the disease. Therefore we conducted a study to evaluate the role of microRNAs (miRNAs) polymorphisms in leprosy. We observed that a polymorphism in miR-146a is associated with the risk to develop leprosy in Brazilians. Based on the analysis of clinical specimens, we found that the genetic variant was correlated with elevated levels of miR-146a and it is also a negative regulator of tumor necrosis factor (TNF), an important inflammatory mediator in the leprosy context. These findings provide tenable evidences that miR-146a is important in the control of gene expression during *M. leprae* infection and also may contribute with leprosy development by controlling TNF levels.

## Introduction

Leprosy is an ancient disease caused by *Mycobacterium leprae*. Once infected, the majority of individuals may clear the bacilli through a natural resistant response. Nevertheless, some patients could develop a latent infection that eventually evolves to one of the clinical symptomatic forms of leprosy. Peripheral nerves Schwann cells and skin macrophages are preferentially invaded, evoking a chronic infection that may take years to become active. Once the disease is established, a range of immune responses occur in spite of *M. leprae* been genetically conserved [1]. The spectral clinical manifestations are classified in a five-group system proposed in the 1960s by Ridley and Jopling [2]. A classic view of predominant Th1 for tuberculoid (TT) pole where a localized form of the disease is observed, in contrast to a major Th2 profile, where a disseminated form, called lepromatous (LL) pole is verified [3]. This classification system also comprises intermediate phenotypes, known as borderline, that interpose those two well characterized poles. Also, a variable percentage of the patients can experience an abrupt inflammatory episodes during the natural course of the disease, which are called type I (reversal) or type II (erythema nodosum leprosum) reactions [4], [5]. Patients at the onset of the episodes exhibit high cytokine levels that are decreased once anti-inflammatory drugs are effective [6]–[8], while genetic association might also be important [9].

Host susceptibility or protection is associated with the complex interaction between environment and genetic background, leading to different outcomes. Several publications aimed to understand the genetic contribution to leprosy risk or protection using different approaches including: twin studies, family-based linkage analysis, candidate gene association and genome wide association studies [10]–[13]. In fact, studies are linking or associating genes that have been generating a compelling amount of evidence to confirm the genetic influence in leprosy outcome. For instance, genes associated with innate immune response, like *TLR1*, *NOD2*, and *PARK2* [11], [14]–[16] or adaptive immune responses, such as *IL10*, *IFNG* and *LTA*/*TNF*/*HLA* have been consistently associated with leprosy [9], [13], [17]–[19].

Recently, microRNAs (miRNAs) have been described as novel regulators of innate and adaptive immune responses, although a few data reported its involvement in leprosy. MiRNA genes are transcribed by RNA polymerase II [20], resulting in a hairpin primary-miRNA (pri-miRNA) that is processed, in a cascade, by different RNAses [21] generating pre-miRNA, and finally the mature miRNA strand facilitating the miR-RISC (RNA-induced silencing complex) assembly [20], [22]. The miRNAs control gene expression at post-translational level by pairing with 3′-untranslated regions [23] leading to mRNA cleavage or translational repression [24]. Given that, it is possible to assume that the presence of polymorphisms along double-stranded sequences can affect miRNA expression and gene silencing [25]. Genetic variants in miRNA precursors, miR-196a-2 (rs11614913 C>T) and miR-146a (rs2910164 G>C) have been associated with cancer and tuberculosis [26]–[30]. Here, we conducted a case-control and a family-based study to test these miRNA SNPs with leprosy susceptibility. Further, we performed functional studies using cell cultures and biopsies from skin and nerves to investigate miRNA mature expression form to define a genotype-phenotype correlation.

## Materials and Methods

### Subjects for the genetic study

The case-control study includes a total of 1,098 individuals from Rio de Janeiro; of these, the 491 patients were recruited from the Souza Araújo outpatient unit, located at Fundação Oswaldo Cruz (FIOCRUZ), Rio de Janeiro, Brazil. The data for 607 controls was obtained from a bone marrow donors' bank in Rio de Janeiro comprising of samples from local healthy individuals. A detailed presentation of this population has been described in Table S1 and elsewhere [16], [31]. A replication population was also tested. Subjects for the family-based study were enrolled from Duque de Caxias, a hyper endemic city from the Rio de Janeiro state (Table S2). This population exhibited 97 nuclear families (426 subjects) [31]. All patients were routinely diagnosed according to Ridley and Jopling criteria (1966). Also, we adopted the World Health Organization (WHO) classification for treatment purposes, and patients were classified as paucibacillary/PB (including TT and borderline-tuberculoid) and multibacillary/MB (including LL, borderline-lepromatous and borderline-borderline). Population characteristics according to the WHO classification and reactional status are summarized in Table S1 and S2. All patients signed an informed consent and this project was approved by the institutional ethics committees from the involved institutions.

### Nerve and skin biopsy specimens

Nerve biopsy samples were obtained at Souza Araújo outpatient unit. A detailed description of nerve samples and clinical forms was previously published [32]. To perform the correlation of TNF mRNA expression with miR-146a genotype we used 33 nerve samples (19 diagnosed with leprosy and 14 with other neuropathies). Among these specimens, we were able to determine miR-146a expression in 12 samples from leprosy patients and 7 from other peripheral neuropathies. In the group of neuropathies other than leprosy, our clinicians were able to accurately diagnose three out of 7 patients. Among those there was: chronic inflammatory demyelinating polyneuropathy (CIDP, n = 2); and one case of systemic lupus erythematous. All undiagnosed patients returned to their neurological clinic for follow-up.

Skin biopsies were obtained from patients who live in Rondonópolis (Mato Grosso State, Brazil), enrolled and diagnosed by professionals from Instituto Lauro de Souza Lima (Bauru city, São Paulo State, Brazil). These specimens comprise 54 skin samples, amongst which 17 patients were diagnosed as MB [borderline borderline (BB) = 10, borderline lepromatous (BL) = 2, LL = 5; distributed as 3 women and 14 men; mean age: 41.6±9.6]. Thirty seven patients were classified as PB [borderline tuberculoid (BT) = 21 and TT = 16; distributed as17 women and 20 men; mean age: 43.9±16.9].

### Ethics statement

The sample collection and procedures described in this work were approved by the Oswaldo Cruz Foundation (FIOCRUZ) and Instituto Lauro de Souza Lima (ILSL) ethics committees. All patients or their parents/guardians signed a written informed consent (IRB protocol - Fiocruz 151/01 and ILSL 172/09).

### Mycobacteria

*M. bovis* BCG Moreau strain (obtained from Fundação Ataulpho Paiva, Rio de Janeiro, Brazil) was cultured, for about 2 weeks, in Middlebrook 7H9 (Invitrogen, Carlsbad, CA) containing 0.02% glycerol and enriched with 10% ADC Middlebrook and 0.5% Tween-80 at 37°C as described elsewhere [32]. Live *M. leprae* from Instituto Lauro de Souza Lima (Bauru, São Paulo) was aseptically cultured in footpads of athymic NU/NU mice, purified and enumerated using methods described previously [33]–[35]. All infections experiments with live *M. leprae* were conduct at 33°C. A portion of live *M. leprae* was irradiated with ionizing radiation 10 kiloGray (Acelétrica Ltda).

### Cell cultures and infection

THP-1 cells were purchased from American Type Culture Collection (ATCC, Rockville, EUA) and cultivated with RPMI-1640 (LGC Biotecnologia, Brazil) supplemented with 2 mM L-Glutamine, 100 U/mL penicillin, 100 µg/mL streptomycin and 10% heat-inactivated FBS (HyClone Laboratories, Canada) at 37°C, 5% CO_2_. Before infection, cells (5×10^5^/well) were differentiated into macrophage-like cells (mTHP-1) using 80 nM phorbol 12-myristate 13-acetate (PMA, Sigma-Aldrich) for 24 h. Then, mTHP-1 were washed with PBS (1×), which was replaced by fresh antibiotics-free medium. Subsequently, stimulation (3, 24, and 48 h) was performed with irradiated or live *M. leprae* (Multiplicity of Infection - MOI 10∶1, 100∶1) at 33°C. After infection, total RNA was extracted as described below.

PBMC from healthy donors were collected in K_3_EDTA-tube (Labor Import Com. Imp. Exp. Ltda, Brazil), and isolated by Ficoll-Hypaque density gradient. After centrifugation (2,500 rpm, 30 min, 25°C), the interface containing mononuclear cells monolayer was collected, washed twice with PBS 1×, and cultivated in RPMI 1640 (LGC Biotecnologia, Brazil) supplemented with 10% human A/B RH^+^ serum (Sigma-Aldrich).

### ELISA

After isolation, PBMC were carefully selected according to miR-146a genotype (rs2910164 G>C), as described below. Cells were infected with BCG Moreau strain (MOI 10∶1) for 24 hours at 37°C. BCG was used as a surrogate model for *M. leprae* infections since it is a better inducer of TNF and also because this mycobacteria is able to induce miR-146a expression in vitro (data not shown). The supernatant was collected and detection to TNF levels was evaluated by Enzyme-linked immunosorbent assay kit DuoSet (R&D Systems, EUA) according to the manufacturer's protocol. Samples optical density (OD) was taken at 450 nm and estimated based on a standard curve, ranging (15.6–1000 pg/mL). Measurements were performed in duplicate.

### Nucleic acid extraction

Genomic DNA for the genetic study was extracted from peripheral blood or directly from PBMCs aliquots according to salting-out method as described [36]. RNA, and then DNA, from skin and nerve biopsy specimens were extracted using Trizol (Invitrogen) according to the manufacturer's instructions [37]. After DNA extraction, all samples were genotyped for SNPs as described below. Total amount of nucleic acids and purity were measured at NanoDrop ND-1000 (Thermo Scientific) instrument. The quality inspection of RNA was tested using agarose gel electrophoresis (1.2%) of 200 ng of SYBR Green II-stained RNA visualized at a transiluminator system (L-Pix Touch, *Loccus Biotecnologia*).

### MiRNAs genotyping and gene expression analysis by PCR

Allelic discrimination was performed using TaqMan Genotyping Assay (Applied Biosystems, CA, USA) for miR-196a-2 (rs11614913 C>T), miR-146a (rs2910164 G>C), miR-125a (rs12975333 G>T) and miR-223 (rs34952329 *>T) SNP. DNA (10–50 ng) amplification was performed in a final volume of 5 µL (2.5 µL of the TaqMan Genotyping Master Mix (Applied Biosystems), 0.125 µL of the TaqMan primers and probes). For miRNA expression analysis we performed a pooled-RT by using a set of specific stem-loop primers for each target (miR-146a, RNU44, RNU48) as indicated by the manufactures (TaqMan, Applied Biosystems). Briefly, non-denatured total RNA (200 ng) was incubated with RT primer pool (0.02×), dNTP (2 mM), Superscript III (10 U/µL, Invitrogen), RNase inhibitor (0.253 U/µL, Invitrogen) and first strand buffer (1×) in a final volume of 15 µL. The cDNA obtained was diluted (1∶6) and 2 µL were subjected to real-time PCR reaction, at a final volume of 10 µL. For RPL13a and TNF expression, total RNA (500 ng) was reversed transcribed following Superscript III manufacturer's instruction (Invitrogen). Then, 5 µL of diluted cDNA (1∶5) was amplified by real time PCR using SYBR Green PCR Master Mix (1×) and primers (0.5 µM) at the final volume of 20 µL. Both genotyping and miRNA expression were run on a StepOne Plus thermocycler detection system (Applied Biosystems). Specifically for TNF mRNA expression in nerve biopsies, data was retrieved from previous experiment and reanalyzed stratifying patients according to genotypes. Quantitative RT-PCR was performed using Biomark multiplex assays (Fluidigm, CA) as previously described [32].

### Statistical analysis

After genotyping, we performed the Hardy-Weinberg equilibrium (HWE) analysis by chi-square tests. Then, we determined the genotypic, allelic and minor allele carriers frequencies, in order to perform comparisons between case and control groups. Genotypes and alleles with higher frequency were taken as baseline. The measure of allelic and genotypic association with leprosy was estimated by the Odds Ratio (OR) values generated after the application of a logistic regression model as described in detail elsewhere [31]. We also assessed an OR value adjusted for sex, ethnicity and age-at-onset for all comparisons evaluated (leprosy *per se*, subgroup PB–MB, reaction *per se*, and types of leprosy reactions). We also performed a case-control analysis using age as a categorical variable, for that analysis we adjusted for sex and ethnicity. Case-control study statistical analysis was performed using the packages *genetics* and *coin* from open source software R version 2.12.2 (available at http://www.R-project.org/). MiR-146a allele-dose effect (*GC/CC*) was determined by the Cochran–Armitage trend test. The analysis of the family-based transmission/disequilibrium test (TDT) was performed with FBAT software, version 2.0.2c. The TDT allows exploring if miR-146a *C*-allele is transmitted, from heterozygous parents to its affected child [38]. The amount of transmitted alleles was determined in the software Haploview [39]. We first performed the TDT analysis considering all affected child, regardless of their clinical form. A second analysis was conducted to verify the allele transmission to affected child according to their PB/MB status. To test for statistical significance among subgroup analysis we performed heterogeneity testing determined by Cochran's Q statistic.

Gene expression statistical analyses were done using Prism 5 (GraphPad software). Two-tailed Mann-Whitney *t*-test was applied for two sample group comparisons. For multiple testing, Kruskal-Wallis test was used followed by Dunn's post-test. Data is presented as mean ± SEM, except for ELISA (median). The value of p<0.05 was taken as statistically significant.

## Results

### MiR-146a (rs2910164) *C-*allele is associated with risk to leprosy *per se* and clinical forms

Allelic, genotypic and carrier frequencies were determined in both cases and controls and they did not deviate from HWE for SNPs miR-146a and miR-196a-2 (Table 1). The other two tested SNPs (miR-125a and miR-223) proved not polymorphic in our population. No association was detected for miRSNP-196a-2 genotypic or allelic frequencies with leprosy *per se*. Nevertheless, the polymorphisms of miR-146a gene have a susceptibility effect to leprosy *per se* for genotypes (*GC* OR_adjusted_ = 1.44; p = 0.04 and *CC* OR_adjusted_ = 2.18; p = 0.0091). This effect was also observed for allelic frequencies and *C*-allele carriers (OR_adjusted_ = 1.47; p = 0.03 and OR_adjusted_ = 1.56; p = 0.008; respectively). The genotypic OR values prompted us to investigate if it was directly proportional to the *C*-allele presence in each genotype (allele-dose effect), which was confirmed by applying the Cochran–Armitage trend test (χ^2^ = 96.6, p = 2.2×10^−16^). Interestingly, when we evaluated the influence of age-at-leprosy diagnosis in the association effect of miR-146a, we found a stronger effect in the subgroup correspondent from 25 to 34 years/old (Figure S1 and Table S3). Furthermore, we performed a comparison between controls and clinical forms, as stratified as PB and MB (Table S4). Once again, we could not find any association with miR-196a-2. Nonetheless, for both comparisons (PB vs. control or MB vs. control) we observed the risk association of *C-*allele for miR-146a, which was more prominent in the PB group. Significance levels were maintained in different comparison levels in PB subgroup versus controls rather than MB subgroup (Table S4). But, the heterogeneity test demonstrated no statistical significance (p-value = 0.40)

**Table 1:** Genetic association of the miRSNP-196a-2 and miRSNP-146a in the Rio de Janeiro sample: a case-control study of leprosy *per se*.

SNP	Genotype/Allele	Case	Control	OR (95% CI; p Valor)	OR (95% CI; p Valor)*
**miR-196a-2 (rs11614913)**	CC^b^	237 (0.48)	272 (0.47)	-	-
	CT	203 (0.41)	251 (0.44)	0.93 (IC = 0.72–1.2; p = 0.5655)	1.02 (IC = 0.71–1.46; p = 0.90)
	TT	51 (0.1)	52 (0.09)	1.13 (IC = 0.74–1.72; p = 0.5841)	1.18 (IC = 0.64–2.19; p = 0.59)
	Total	491	575		
	C-Allele^b^	677 (0.69)	795 (0.69)	-	-
	T-Allele	305 (0.31)	355 (0.31)	1.01 (IC = 0.78–1.31; p = 0.9468)	1.06 (IC = 0.73–1.54; p = 0.75)
	T-Carriers	254	303	0.96 (IC = 0.76–1.22; p = 0.7533)	1.05 (IC = 0.74–1.48; p = 0.78)
**miR-146a (rs2910164)**	GG^b^	184 (0.38)	330 (0.54)	-	-
	GC	246 (0.5)	242 (0.40)	**1.82 (IC = 1.42–2.35; p = 3.28×10^−06^)**	**1.44(IC = 1.02–2.05; p = 0.04)**
	CC	58 (0.12)	35 (0.06)	**2.97 (IC = 1.88–4.69; p = 2.93×10^−06^)**	**2.18 (IC = 1.21–3.93; p = 0.0091)**
	Total	488	607		
	G-Allele^b^	614 (0.63)	902 (0.74)	**-**	**-**
	C-Allele	362 (0.37)	313 (0.26)	**1.7 (IC = 1.32–2.21; p = 0.0001)**	**1.47 (IC = 1.03–2.11; p = 0.03)**
	C-Carriers	304	277	**1.97 (IC = 1.54–2.51; p = 4.68×10^−08^)**	**1.56 (IC = 1.12–2.18; p = 0.008)**

Population counts are shown as N (frequency).

*Adjusted for sex, ethnicity and age.

b Genotype or allele used as baseline.

Global p-value to miRSNP-146a p = 0.002. Trend test: miRSNP-146a *C*-allele, χ^2^ = 96.6; p = 2.2^−16^/case-control comparison. HWE of control population: miRSNP-196a-2; χ^2^ = 0.2; p = 0.65 and miRSNP-146a; χ^2^ = 0.95; p = 0.34.

A confirmation of the genetic finding for miR-146a was observed after TDT analysis (Table 2). Considering leprosy *per se*, twenty-eight over 41-affected informative patients, received the *C*-allele indicating its over-transmission (p = 0.003). When we stratified affected individuals according to their PB or MB classification, the TDT for PB patients revealed sixteen over twenty one-affected transmissions (p = 0.01). The number of transmissions of the *C*-allele for MB affected patients were not significant (p = 0.23) (Table S5). These data confirm that miR-146a, in our replication sample, is associated with leprosy *per se*, however we could not provide sufficient evidence to infer subtype specificity.

**Table 2:** Family Based Association test of 97 family cores, n = 426 individuals from Duque de Caxias city.

miRSNP-146a	Transmitted	Not transmitted	Allele Frequency	Z Test	p-Value
**G**	13	28	0.66	−2.979	0.003
**C**	**28**	13	0.34	2.979	**0.003**

### MiR-146a (rs2910164 G>C) SNP was not associated with leprosy reaction

In order to evaluate if those miRSNPs could be associated with leprosy reaction episodes, we subdivided only our patient group in (1) controls, patients without occurrence of reactional episodes and (2) cases, patients who exhibited only one type of leprosy reactional (LR) episodes (erythema nodosum leprosum, ENL or reverse reaction, RR). Those who have experienced both episodes were excluded from analysis. We could not observe any association between miRSNP-196a-2 and leprosy reactions (data not shown). Although we found that *CC* genotypes showed borderline association with protection to leprosy reactions (Table S6), and ENL as outcome, no statistical significance was found after adjustment for the covariates gender, ethnicity and age (Table S7).

### Live *M. leprae*, but not irradiated *M. leprae*, was able to induce miR-146a expression

**Figure 1:**
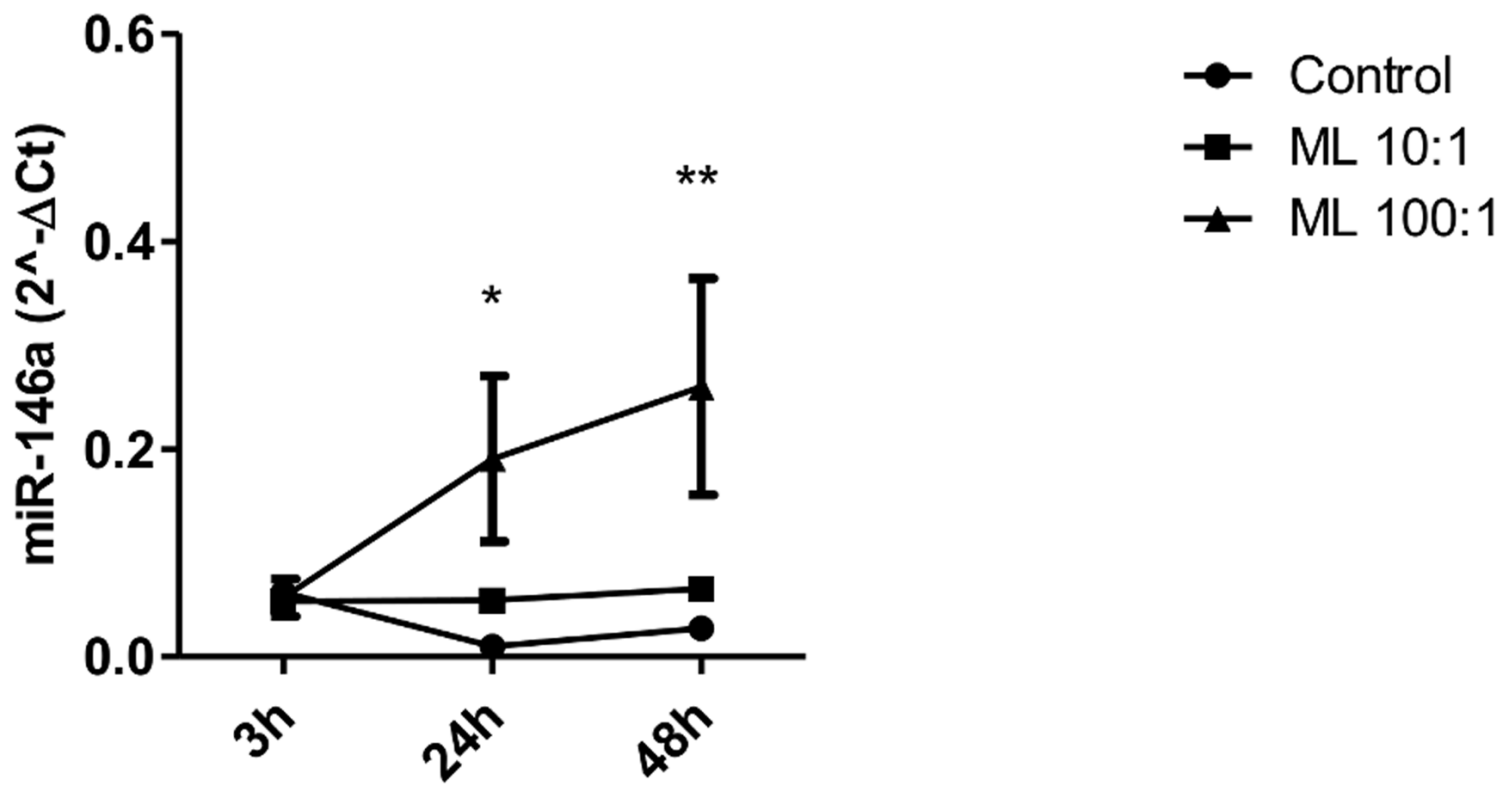
MiR-146a expression in cells exposed to *M. leprae*. Macrophage-like THP-1 cells (5×10^5^) were infected with live *M. leprae* (MOI 10∶1, 100∶1) for 3, 24 and 48 h at 33°C. RNA was extracted and a real-time stem-loop RT-PCR was performed using RNU48 to normalize. Data show mean ± SEM (*p<0.05 relative to 24 h control, **p<0.05 relative to 48 h control). Results represent four independent experiments.

Previous results have shown that specific pathways associated with pro-mycobacterial profiles that reprogram cellular environment to establish a suitable niche for bacterial survival are dependent on *M. leprae* viability [40]. So, we asked whether the functional role of miR-146a was dependent on live *M. leprae*. Irradiated *M. leprae* did not induce miR-146a expression (data not shown), on the other hand, live *M. leprae* infection for 3, 24 and 48 h at two different MOIs (10∶1 and 100∶1) induces miR-146a expression at 100 bacilli per cell (Figure 1). MiR-146a expression started at 24 h and was sustained until 48 h of infection.

### Mature miR-146a expression is differentially expressed in nerves from leprosy patients and it is affected by host genotype

**Figure 2:**
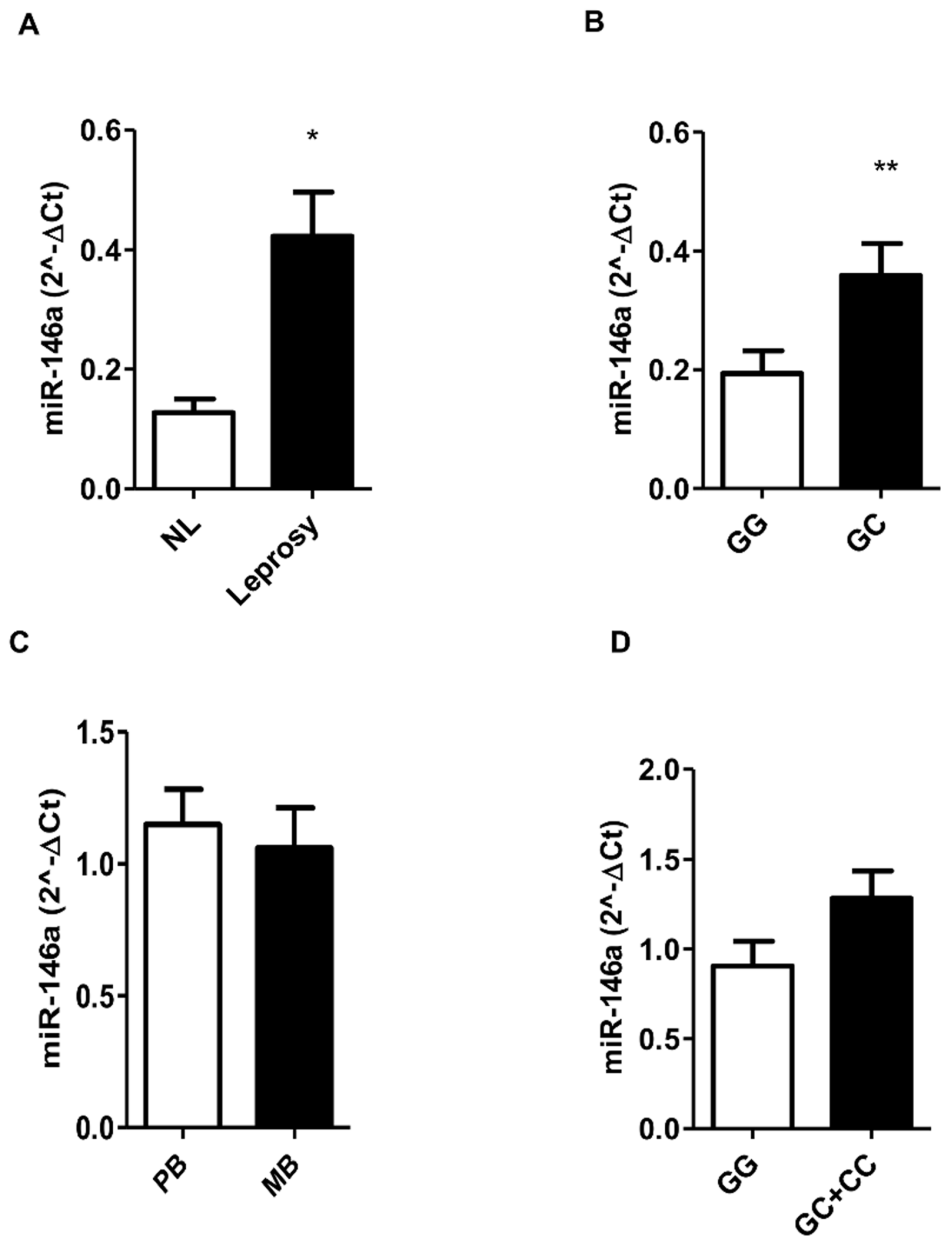
MiR-146a expression in nerve and skin biopsies, and according to patients' genotype (rs2910164). MiR-146a mature form expression was analyzed in nerve (A, B) and skin (C, D) specimens by real-time stem-loop RT-PCR after total RNA extraction. RNU48 expression was used to normalization. (A) Analysis was performed comparing the levels of miR-146a expression in nerve biopsies (NL = not leprosy, n = 7/Leprosy, n = 12). (B) MiR-146a expression stratified by rs2910164/G>C genotype, *GG* (n = 8) and *GC* (n = 7). The data show mean ± SEM (*p = 0.0099; and **p = 0.0401) (C) Results show neither difference of miR-146a expression in skin biopsies according to clinical forms (PB = 37 and MB = 17), nor by genotype stratification (*GG* = 10; *GC* = 22; *CC* = 4) (D).

So far, we observed an associated SNP in a gene that was being up-regulated by *M. leprae* infection. Then, we decided to explore miR-146a expression in skin and nerve biopsy samples trying to correlate genetic, clinical and biological findings. Initially, we determined miR-146a levels in nerve leprosy patients (L) and performed a comparison with biopsies from patients with non-leprous (NL) neuropathies. We found that miR-146a is more expressed in leprosy nerve biopsies group than in NL biopsies (Figure 2A). The examination of miR-146a expression according to host genotype revealed that carriers of *C-*allele were able to produce high levels of the mature miRNA (Figure 2B) in nerves. We determined miR-146a levels in skin biopsies from leprosy patients and performed a comparison between the MB and PB groups. Our results showed that biopsies from patients in both clinical forms express moderate-to-high levels of this miRNA, but no difference between MB and PB was detected (Figure 2C). Stratification according to Ridley and Jopling (R&J) clinical forms (LL, BL, BB, BT and TT) did not show any differences in miR-146a levels (data not shown). Furthermore, stratification by the risk allele (*C*-carriers) showed a tendency of augmented miR-146a expression in skin biopsies, although not significant (Figure 2D). Comparisons indicate that miR-146a *C*-allele seems to induce higher levels of miR-146a, which was also increased in leprosy patients, although no clustering was observed between clinical forms.

### TNF expression and secretion was influenced by rs2910164 miR-146a genotypes

**Figure 3:**
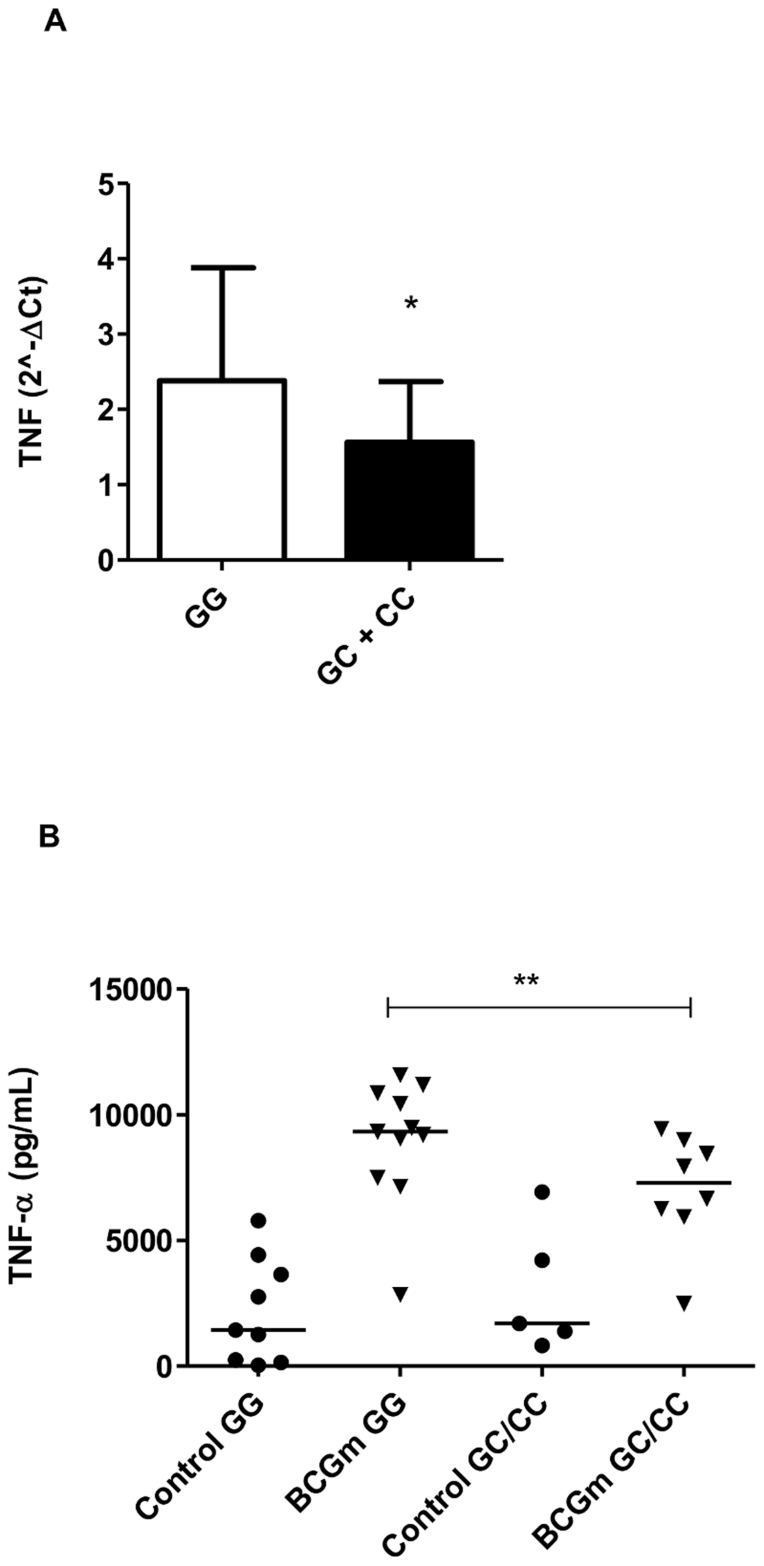
TNF expression and secretion according to the host genotypes (rs2910164). (A) TNF expression from nerve biopsies was assessed by qRT-PCR after RNA isolation. Standardized expression values are shown. Control genotype (*GG*) = 12 and risk genotype (*GC*+*CC*) = 15. Data show the mean ± SEM (*p = 0.0453). (B) To assess TNF secretion, PBMC (3×10^6^ cells) were infected with BCG Moreau (BCGm) at a MOI 10∶1 for 24 h at 37°C. Supernatant was collected and TNF concentration (pg/mL) was estimated by ELISA. TNF levels produced by infected cells were significantly lower in *C-*allele carriers (risk variant) when compared with infected cells with *GG* genotype. Lines represent the median of each group and the dots represent individual PBMC donors. (**p = 0.0352).

It was previously reported that miR-146a negatively regulates cytokines in primary peritoneal macrophages of mice, such as TNF [41]. Therefore, at this point, our hypothesis was whether the polymorphism associated with risk (*C*-allele) correlates to lower levels of TNF. For this, we analyzed the expression of TNF in nerve biopsies [32] and stratified according to the genotypes. As shown in Figure 3A, the presence of *C*-allele is associated with a reduction of TNF expression (p = 0.045), irrespective the disease type (L or NL). Then, we tested if miR-146a genotypes could influence TNF secretion. For that purpose, the cells were either left uninfected or infected with BCG Moreau and we compared infected groups with different genotypes. As shown in Figure 3B, the presence of the *C-*allele was related with less TNF secretion when comparing it to its control, GG-infected genotype (p = 0.0352).

## Discussion

In this study we showed that a SNP in miR-146a (rs2910164G>C), located in a cytokine cluster (5q31) associated with autoimmunity [42], [43] and Crohn's disease [44], was associated with leprosy susceptibility. Interestingly, live *M. leprae* up regulates this miRNA and carriers of the risk allele were also expressing more miR-146a. Furthermore, we were able to correlate lower levels of TNF with the presence of the risk allele. We also selected other two candidate miRNA SNPs (miR-125a, miR-223) previously identified as associated with regulation of immune responses [45], [46], but neither were polymorphic in Brazilians. The miR-196a-2 were chosen based on their involvement in Crohn's disease [47]. Nonetheless, we could not find any association between miR-196a-2 and leprosy although a previous report provided evidence to a common genetic fingerprint in Leprosy and Crohn's disease [48].

It was reported that miR-146a (rs2910164) *GC* polymorphisms plays an important role in papillary thyroid carcinoma while *CC* genotype are linked with risk and the reduction of survival in patients with glioma [49]. Controversial studies concerning susceptibility to cancer were investigated by a meta-analysis. They could not find a pattern between the SNP and the tumor type, conversely, the study pinpointed that there is an association between *GG* variant genotypes and increased risk of cancer among Asians [50], maybe reflecting the heterogeneity of the disease. Considering mycobacteria infections, it was demonstrated that the *G-*allele has an association with pulmonary tuberculosis in different directions in Han (protection) and Tibetan (risk) populations [51]. Here, we provide consistent evidence of G>C miRSNP-146a associated with leprosy among Brazilians. Using two different study designs, case-control and family-based, we found that the *C*-allele was strongly associated with susceptibility to leprosy *per se* and age-at-diagnosis was an important adjustment for the association, which was also suggested previously in leprosy [52], [53]. For all case-control comparisons, we tested the miRSNP-146a association considering sex and ethnicity with or without (data not shown) age as covariate, but the results remain unaltered after the inclusion of age-at-diagnosis correction. However, considering age subsets independently, a stronger association in the early-onset leprosy (25–34 years/old) was detected. This last observation is consistent with the idea that the early-onset may reflect a stronger genetic effect [17], [52], [54]. Curiously, the genetic design using leprosy reactions as outcome suggested an association between *CC* genotypes and LR protection towards protection, although not statistically significant after correction considering or not the covariate age.

MiR-146a mature form contributes to the reduction of TNF synthesis by down-regulation of adapter molecules IRAK1/TRAF6 through 3′UTR matching [55]. In THP-1 ectopically super-expressing miR-146a, Boldin and coworkers described that the exacerbated immune response was down-regulated by the reduction of TNF and IL-6 levels. Also, they found an uncontrolled autoimmune profile in miR-146a^−/−^ knockout mice, as the animals were hyper-responsive to LPS challenge, producing high levels of those pro-inflammatory cytokines TNF, IL-6 [41] that was also in agreement with previous reports [56], [57] and our results here. It was recently shown that *M. bovis* BCG induces miR-146a expression and regulates TNF levels [58]. In our model, only live *M. leprae* was able to stimulate miR-146a expression. A recent paper from Siddle and colleagues, identified some SNPs in miRNA genes as markers of expression of quantitative trait loci (eQTL) in dendritic cells infected with *M. tuberculosis* [54], [59]. In fact, it has been proposed that SNPs along the strands that generate miRNAs can have great impact on both biogenesis of mature miRNA as well as the gain or loss of function of a particular miRNA [25], [60]. The miRSNP-146a is localized in the precursor strand and involves a shift of G∶U pair to C∶U mismatch. Jazdzewski showed that miR-146a expression was lower in the presence of the *C*-allele when compared to the *G*-allele [27], [28]; confirmed by others reports on cancer [61], [62]. In our analysis, miR-146a expression in nerve biopsies from leprosy patients revealed a different pattern: *C*-allele carriers are related with the high levels of the mature miR-146a. In agreement with our findings, it was shown by Kogo and colleagues that miR-146a was highly expressed in carriers of *CC* genotype than *GG* in both healthy and tumor tissues from patients with gastric cancer [63]. Also, in lupus a study showed that the presence of the *C*-allele correlates with increased expression of the mature miR-146a [64]. Nevertheless, we did not observe differences in skin biopsies from PB and MB patients. Perhaps, in this case, it seems that rs2910164 G>C SNP might impact miR-146a expression very early in the progression from latent infection to active disease, since pure neural form could be considered an earlier stage of the leprosy development. We could hypothesize that in the early stages of progression towards active disease, miR-146a expression in the macrophages/dendritic cells may be differentially regulating cytokine secretion and the emergence of T cell specific subpopulations precipitating disease outcome [65]. This controversy in the literature suggests that the presence of this SNP G>C (rs2910164), and maybe others, may govern the expression of mature miRNAs.

Recently it has been shown that miR-21 targeting CYP27b1, an enzyme that convert the vitamin D pro-hormone to its active form, inhibits the microbicidal vitamin D dependent-pathway [66]. Also, the same study showed that miR-146a was the second most differentially expressed miRNA in lepromatous leprosy skin biopsies [66], although in our hands, with a high number of samples we detected no difference between the clinical forms of the disease.

In summary, we demonstrated the genetic association between miR-146a *C*-allele with leprosy susceptibility. Our data also suggest that miR-146a was overexpressed in leprosy biopsies and also produced by mTHP-1 infected with live *M. leprae*. Subjects carrying the risk allele also express high levels of miR-146a which correlates with lowest levels of TNF as readout of the inflammatory responses.

## Supporting Information

Figure S1 **The impact of “age-at-diagnosis” in miRSNP-146a association with leprosy** ***per se*** **susceptibility using age as a categorical variable.** The case-control analysis was performed considering different age subsets independently. We consider subsets of age-at-diagnosis less than 25 years old (yrs), from 25 to 34 yrs, from 35 to 44 yrs, from 45 to 54 yrs and equal or older than 55 yrs. The results showed a main effect of susceptibility association in the subset ranging 25 to 34 yrs, considering the homozygous genotype, *C*-allele and *C*-carriers. Also, we observed an association of heterozygous genotype and *C*-carriers in the 35 to 44 yrs subset. The *GG* genotype or *G*-allele was used as baseline. The results showed OR values adjusted for sex and ethnicity, age was used as categorical variable (*p-value<0.05 and **p-value = 0.06). A detailed description of “age-at-diagnosis” subset populations can be found in supplemental table S3.
(TIF)

Table S1 **Characteristics of the population included in the case-control study.**
(DOCX)

Table S2 **Characteristics of the population included in the TDT study.**
(DOCX)

Table S3 **Genotype counts for miRSNP-146a in the Rio de Janeiro case-control group stratified according to age-at-leprosy.**
(DOCX)

Table S4 **Genetic association of the miRSNP-196a-2 and miRSNP-146a in Rio de Janeiro population: a case-control study of leprosy outcomes (MB and PB).**
(DOCX)

Table S5 **Family-Based Association test of leprosy outcome (PB and MB) from Duque de Caxias city population.**
(DOCX)

Table S6 **Genetic association of miRSNP-146a in Rio de Janeiro population: a case-control study of leprosy reaction** ***per se*** **.**
(DOCX)

Table S7 **Genetic association of miRSNP-146a in Rio de Janeiro population: a case-control study of leprosy reaction type as outcomes (RR and ENL).**
(DOCX)

Checklist S1 **STROBE checklist.**
(DOCX)
