# Supplementary figures and images for: Pre-miR-146a (rs2910164 G>C) Single Nucleotide Polymorphism Is Genetically and Functionally Associated with Leprosy
Source: PLoS Negl Trop Dis. 2014 Sep 4;8(9):e3099. doi: 10.1371/journal.pntd.0003099 (PMC4154665; doi:10.1371/journal.pntd.0003099)

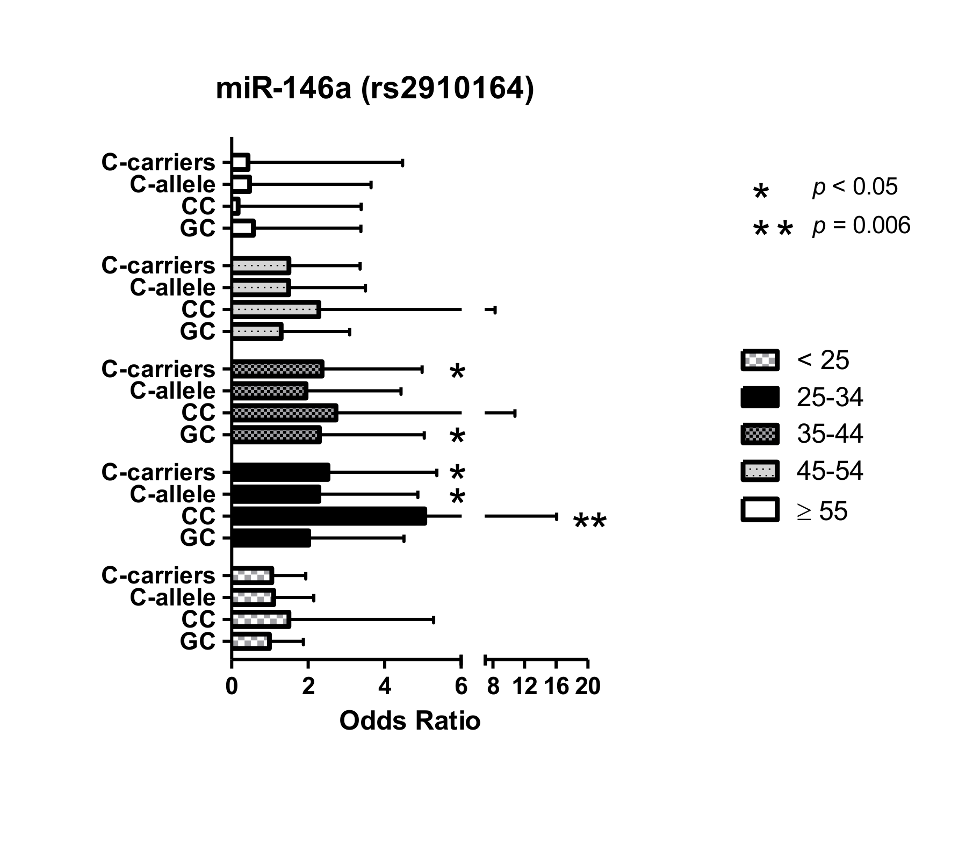

Supplement: Figure S1 — The impact of “age-at-diagnosis” in miRSNP-146a association with leprosy per se susceptibility using age as a categorical variable. The case-control analysis was performed considering different age subsets independently. We consider subsets of age-at-diagnosis less than 25 years old (yrs), from 25 to 34 yrs, from 35 to 44 yrs, from 45 to 54 yrs and equal or older than 55 yrs. The results showed a main effect of susceptibility association in the subset ranging 25 to 34 yrs, considering the homozygous genotype, C-allele and C-carriers. Also, we observed an association of heterozygous genotype and C-carriers in the 35 to 44 yrs subset. The GG genotype or G-allele was used as baseline. The results showed OR values adjusted for sex and ethnicity, age was used as categorical variable (*p-value<0.05 and **p-value = 0.06). A detailed description of “age-at-diagnosis” subset populations can be found in supplemental table S3. (TIF) [file pntd.0003099.s001.tif]
