# Supplementary material for: Pre-miR-146a (rs2910164 G>C) Single Nucleotide Polymorphism Is Genetically and Functionally Associated with Leprosy
Source: PLoS Negl Trop Dis. 2014 Sep 4;8(9):e3099. doi: 10.1371/journal.pntd.0003099 (PMC4154665; doi:10.1371/journal.pntd.0003099)
Supplement: Table S1 — Characteristics of the population included in the case-control study. (DOCX) [file pntd.0003099.s002.docx]

| Table S1.Characteristics of the population included in the case-control study. | | | | | | | |  | |
| --- | --- | --- | --- | --- | --- | --- | --- | --- | --- |
|  | | **Case-control study population** | | ***WHO classification*** | | ***leprosy reactions*** | | | |
|  | | **Cases** | **Controls** | **MB** | **PB** | **RR*** | **ENL*** | **None LR** | |
| Age (mean ± SD) | | 39.4±16.8 | 33.5 ± 9.6 | 38.2 ± 16.4 | 41.1 ± 17.2 | 42.2 ± 16.8 | 34 ± 14.2 | 40.3 ± 17.4 | |
| Sex | |  |  |  |  |  |  |  | |
| *Female* n (frequency) | | 325 (0.36) | 314 (0.45) | 152 (0.28) | 173 (0.5) | 48 (0.41) | 37 (0.28) | 73 (0.46) | |
| *Male* n (frequency) | | 569 (0.64) | 386 (0.55) | 396 (0.72) | 173 (0.5) | 69 (0.59) | 94 (0.72) | 84 (0.54) | |
| Ethnicity | |  |  |  |  |  |  |  | |
| *Caucasoids* n (frequency) | | 438 (0.56) | 393 (0.57) | 266 (0.57) | 172 (0.54) | 49 (0.58) | 41 (0.50) | 63 (0.55) | |
| *Mestizoes* n (frequency) | | 271 (0.34) | 195 (0.28) | 156 (0.33) | 115 (0.36) | 26 (0.30) | 31 (0.37) | 43 (0.38) | |
| *Blacks* n (frequency) | | 79 (0.10) | 105 (0.15) | 46 (0.10) | 33 (0.10) | 10 (0.12) | 11 (0.13) | 8 (0.07) | |
| Abbreviations: MB, multibacillary; PB, paucibacillary; RR, reverse reaction; ENL, erythema nodosum leprosum and LR, leprosy reactions  * patients that have experienced only one kind of leprosy reaction episodes | | | | | | | | | |
