# Supplementary material for: Pre-miR-146a (rs2910164 G>C) Single Nucleotide Polymorphism Is Genetically and Functionally Associated with Leprosy
Source: PLoS Negl Trop Dis. 2014 Sep 4;8(9):e3099. doi: 10.1371/journal.pntd.0003099 (PMC4154665; doi:10.1371/journal.pntd.0003099)
Supplement: Table S2 — Characteristics of the population included in the TDT study. (DOCX) [file pntd.0003099.s003.docx]

| Table S2.Characteristics of the population included in the TDT study. | | |
| --- | --- | --- |
|  | **Affected** | **Unaffected** |
| **Age (mean ± SD**) | 13 ± 7.9 | 39 ± 15.8 |
| **Sex** |  |  |
| *Female* n (frequency) | 92 (0.55) | 103 (0.56) |
| *Male* n (frequency) | 75 (0.45) | 79 (0.44) |
| **Ethnicity** |  |  |
| *Caucasoids* n (frequency) | 70 (0.47) | 61 (0.44) |
| *Mestizoes* n (frequency) | 45 (0.30) | 52 (0.37) |
| *Blacks* n (frequency) | 34 (0.23) | 26 (0.19) |
| **WHO classification** |  |  |
| *Paucibacillary* n (frequency) | 68 (0.49) | -- |
| *Multibacillary* n (frequency) | 69 (0.50) | -- |
