# Supplementary material for: Pre-miR-146a (rs2910164 G>C) Single Nucleotide Polymorphism Is Genetically and Functionally Associated with Leprosy
Source: PLoS Negl Trop Dis. 2014 Sep 4;8(9):e3099. doi: 10.1371/journal.pntd.0003099 (PMC4154665; doi:10.1371/journal.pntd.0003099)
Supplement: Table S3 — Genotype counts for miRSNP-146a in the Rio de Janeiro case-control group stratified according to age-at-leprosy. (DOCX) [file pntd.0003099.s004.docx]

| Table S3**.** Genotype counts for miRSNP-146a in the Rio de Janeiro case-control group stratified according to age at leprosy. | | | | | | |
| --- | --- | --- | --- | --- | --- | --- |
|  | **< 25 yrs** | **25-34 yrs** | **35-44 yrs** | **45-54 yrs** | **≥55 yrs** |  |
| **Cases** | **103** | **110** | **69** | **40** | **4** |  |
| GG | 46 (0.45) | 58 (0.53) | 44 (0.64) | 20 (0.50) | 1 (0.25) |  |
| GC | 46 (0.45) | 44 (0.40) | 21 (0.30) | 16 (0.40) | 2 (0.50) |  |
| CC | 11 (0.10) | 8 (0.07) | 4 (0.06) | 4 (0.10) | 1 (0.25) |  |
| **Controls** | **92** | **58** | **61** | **72** | **76** |  |
| GG | 47 (0.51) | 21 (0.36) | 26 (0.43) | 30 (0.42) | 34 (0.45) |  |
| GC | 40 (0.44) | 23 (0.40) | 27 (0.44) | 29 (0.40) | 36 (0.47) |  |
| CC | 5 (0.05) | 14 (0.24) | 8 (0.13) | 13 (0.18) | 6 (0.08) |  |
| Population counts are shown as N (frequency). In bold are shown total counts for each subgroup. Yrs= years old | | | | | | |
