# Supplementary material for: Pre-miR-146a (rs2910164 G>C) Single Nucleotide Polymorphism Is Genetically and Functionally Associated with Leprosy
Source: PLoS Negl Trop Dis. 2014 Sep 4;8(9):e3099. doi: 10.1371/journal.pntd.0003099 (PMC4154665; doi:10.1371/journal.pntd.0003099)
Supplement: Table S4 — Genetic association of the miRSNP-196a-2 and miRSNP-146a in Rio de Janeiro population: a case-control study of leprosy outcomes (MB and PB). (DOCX) [file pntd.0003099.s005.docx]

| Table S4. Genetic association of the miRSNP-196a-2 and miRSNP-146a in a case-control population from Rio de Janeiro using MB and PB as outcomes | | | | | | |  |
| --- | --- | --- | --- | --- | --- | --- | --- |
| SNP | **Genotype**  **/ allele** | **Group** | | | **OR**  **(95% CI; p-Value)*** | **OR**  **(95% CI; p-Value)*** | |
|  |  | **MB** | **PB** | **Control** | **PB vs Control** | **MB vs Control** | |
| miR-196a-2  (rs11614913) | CC ^b^ | 140 (0.48) | 97 (0.49) | 272 (0.47) |  |  | |
|  | CT | 117 (0.40) | 86 (0.43) | 251 (0.44) | 1.04 (IC= 0.66-1.64; p= 0.88) | 1.08 (IC= 0.7 -1.65; p= 0.71) | |
|  | TT | 36 (0.12) | 15 (0.08) | 52 (0.09) | 0.79 (IC= 0.34-1.85; p= 0.59) | 1.53 (IC= 0.77-3.03; p= 0.23) | |
|  | Total | 293 | 198 | 575 |  |  | |
|  | C-Allele ^b^ | 397 (0.68) | 280 (0.71) | 795 (0.69) |  |  | |
|  | T-Allele | 189 (0.32) | 116 (0.29) | 355 (0.31) | 0.95 (IC= 0.59-1.55; p= 0.84) | 1.18 (IC= 0.77-1.83; p= 0.44) | |
|  | T-Carriers | 153 | 101 | 303 | 0.99 (IC= 0.64-1.54; p= 0.98) | 1.16 (IC= 0.78-1.73; p= 0.47) | |
| miR-146a  (rs2910164) | GG ^b^ | 118 (0.41) | 66 (0.33) | 330 (0.54) |  |  | |
|  | GC | 143 (0.49) | 103 (0.52) | 242 (0.40) | **1.47 (IC= 0.93-2.33; p= 0.09)** | **1.52 (IC=1.01-2.29; p= 0.05)** | |
|  | CC | 29 (0.1) | 29 (0.15) | 35 (0.06) | **3.08 (IC=1.53-6.20; p= 0.002)** | **1.62 (IC= 0.81-3.24; p= 0.17)** | |
|  | Total | 290 | 198 | 607 |  |  | |
|  | G-Allele ^b^ | 379 (0.65) | 235 (0.59) | 902 (0.74) |  |  | |
|  | C-Allele | 201 (0.35) | 161 (0.41) | 312 (0.26) | **1.70 (IC= 1.08-2.67; p= 0.02)** | **1.37 (IC=0.90-2.09; p=0.0001)** | |
|  | C-Carriers | 172 | 132 | 277 | **1.54 (IC= 1.09-2.18; p= 0.01)** | **1.56 (IC=1.12-2.18; p= 0.008)** | |
| Population counts are shown as N (frequency). *Adjusted for sex, ethnicity and age. ^b^Genotype or allele used as baseline. For both comparisons in miR-146a SNP the global p-value is <0.001. | | | | | | |  |
