# Supplementary material for: Pre-miR-146a (rs2910164 G>C) Single Nucleotide Polymorphism Is Genetically and Functionally Associated with Leprosy
Source: PLoS Negl Trop Dis. 2014 Sep 4;8(9):e3099. doi: 10.1371/journal.pntd.0003099 (PMC4154665; doi:10.1371/journal.pntd.0003099)
Supplement: Table S5 — Family-Based Association test of leprosy outcome (PB and MB) from Duque de Caxias city population. (DOCX) [file pntd.0003099.s006.docx]

| Table S5. Family Based Association test of leprosy outcome (PB and MB) from Duque de Caxias city population | | | | | |
| --- | --- | --- | --- | --- | --- |
| miRSNP-146a | **Transmitted** | **Not transmitted** | **Allele Frequency** | **Z Test** | **p-Value** |
| Paucibacillary: 45 nuclear families, 195 individuals | | | | | |
| G | 5 | 16 | 0.63 | - 2.560 | 0.01 |
| C | **16** | 5 | 0.37 | 2.560 | 0.01 |
| Multibacillary: 41 nuclear families, 193 persons | | | | | |
| G | 6 | 7 | 0.71 | -1.208 | 0.23 |
| C | 7 | 6 | 0.29 | 1.208 | 0.23 |
| Q statistic (heterogeneity) p-value= 0.40 | | | | | |
