# Supplementary material for: Pre-miR-146a (rs2910164 G>C) Single Nucleotide Polymorphism Is Genetically and Functionally Associated with Leprosy
Source: PLoS Negl Trop Dis. 2014 Sep 4;8(9):e3099. doi: 10.1371/journal.pntd.0003099 (PMC4154665; doi:10.1371/journal.pntd.0003099)
Supplement: Table S6 — Genetic association of miRSNP-146a in Rio de Janeiro population: a case-control study of leprosy reaction per se . (DOCX) [file pntd.0003099.s007.docx]

| Table S6. Genetic association of miRSNP-146a in Rio de Janeiro population: a case-control study of leprosy reaction *per se* | | | | | |
| --- | --- | --- | --- | --- | --- |
| **SNP** | **Genotype/**  **allele** | **Case** | **Control** | **OR (95% CI; p-Value)** | **OR (95% CI; p-Value)*** |
| **miR-146a**  **(rs2910164)** | GG^b^ | 117 (0.41) | 53 (0.34) |  | - |
|  | GC | 138 (0.48) | 78 (0.50) | 0.8 (IC= 0.52-1.2; p= 0.31) | 0.77 (IC= 0.47-1.25; p= 0.30) |
|  | CC | 30 (0.11) | 26 (0.17) | **0.52 (IC= 0.28-0.97; p= 0.039)** | 0.54 (IC= 0.27-1.11; p= 0.097) |
|  | Total | 285 | 157 |  |  |
|  | G-Allele^b^ | 372 (0.65) | 184 (0.59) | - | - |
|  | C-Allele | 198 (0.35) | 130 (0.41) | 0.75 (IC= 0.54-1.12; p=0.17) | 0.76 (IC= 0.48-1.2; p= 0.24) |
|  | C-Carriers | 168 | 104 | 0.73 (IC= 0.48-1.09; p= 0.13) | 0.72 (IC= 0.45-1.14; p= 0.16) |
|  |  |  |  |  |  |
| Population counts are shown as N (frequency). *Adjusted for sex, clinical form (BL, LL), relapse and age. ^b^Genotype or allele used as baseline. Global p-value= 0.75 | | | | | |
