# Supplementary material for: Pre-miR-146a (rs2910164 G>C) Single Nucleotide Polymorphism Is Genetically and Functionally Associated with Leprosy
Source: PLoS Negl Trop Dis. 2014 Sep 4;8(9):e3099. doi: 10.1371/journal.pntd.0003099 (PMC4154665; doi:10.1371/journal.pntd.0003099)
Supplement: Table S7 — Genetic association of miRSNP-146a in Rio de Janeiro population: a case-control study of leprosy reaction type as outcomes (RR and ENL). (DOCX) [file pntd.0003099.s008.docx]

| Table S7. Genetic association of miRSNP-146a in Rio de Janeiro population: a case-control study of leprosy reaction type as outcomes (RR and ENL) | | | | | | | | |  |
| --- | --- | --- | --- | --- | --- | --- | --- | --- | --- |
| **miR-146a**  **(rs2910164)** | **Group** | | | **OR (95% CI)**  **p-Value** | | **OR (95% CI)***  **p-Value** | | | |
| **Genotype/**  **allele** | **Control** | **RR** | **ENL** | **RR vs cont.** | **ENL vs cont.** | **RR vs Cont.** | **ENL vs cont.** | | |
| GG^b^ | 53 (0.34) | 44 (0.38) | 56 (0.43) | - | - | - | - | | |
| GC | 78 (0.50) | 59 (0.50) | 63 (0.48) | 0.91 (IC= 0.54-1.54)  p= 0.73 | 0.76 (IC= 0.46-1.26)  p= 0.29 | 0.86 (IC= 0.50-1.50)  p= 0.60 | 0.71 (IC= 0.29-1.74)  p= 0.45 | | |
| CC | 26 (0.17) | 14 (0.12) | 12 (0.09) | 0.65 (IC= 0.30-1.39)  p= 0.27 | **0.44 (IC= 0.2-0.95)**  **p= 0.04** | 0.53 (IC= 0.23-1.21)  p= 0.13 | 0.56 (IC= 0.16-1.98)  p= 0.36 | | |
| Total | 157 | 117 | 131 |  |  |  |  | | |
| G-Allele^b^ | 184 (0.59) | 147 (0.63) | 175 (0.67) | - | - | - | - | | |
| C-Allele | 130 (0.41) | 87 (0.37) | 87 (0.33) | 0.84 (IC= 0.51-1.37)  p= 0.48) | 0.70 (IC= 0.43-1.14)  p= 0.15 | 0.77 (IC= 0.46-1.30)  p= 0.34 | 0.75 (IC= 0.33-1.71)  p= 0.50 | | |
| C-Carriers | 104 | 73 | 75 | 0.85 (IC= 0.51-1.4)  p= 0.51 | 0.68 (IC= 0.42-1.1)  p=0.12 | 0.78 (IC= 0.46-1.3)  p= 0.35 | 0.67 (IC= 028-1.59)  p= 0.37 | | |
| Population counts are shown as N (frequency). *Adjusted for sex, clinical form (BL, LL), relapse and age. ^b^Genotype or allele used as baseline. Global p-value: RR vs. control p= 0.9666 and ENL vs. control p= 0.4083 | | | | | | | |  |  |
